# School-based screening for psychiatric disorders in Moroccan-Dutch youth

**DOI:** 10.1186/s13034-015-0045-8

**Published:** 2015-05-13

**Authors:** Marcia Adriaanse, Lieke van Domburgh, Barbara Zwirs, Theo Doreleijers, Wim Veling

**Affiliations:** Department of Child and Adolescent Psychiatry, VU Medical Centre, PO Box 303, 1115 ZG Duivendrecht, The Netherlands; Parnassia Academy, Parnassia Psychiatric Institute, Oude Parklaan 123, 1901 ZZ Castricum, The Netherlands; Department of Research and Development, Intermetzo, PO Box 94, 7200 AB Zutphen, The Netherlands; Department of Criminology, Leiden University, Steenschuur 25, 2311 ES Leiden, The Netherlands; Department of Psychiatry, University Medical Centre Groningen, University of Groningen, PO Box 30001, 9700 RB Groningen, The Netherlands

**Keywords:** Screening, Questionnaires, Children, Adolescents, Cross-cultural, Externalizing disorders, Internalizing disorders

## Abstract

**Background:**

While ethnic diversity is increasing in many Western countries, access to youth mental health care is generally lower among ethnic minority youth compared to majority youth. It is unlikely that this is explained by a lower prevalence of psychiatric disorders in minority children. Effective screening methods to detect psychiatric disorders in ethnic minority youth are important to offer timely interventions.

**Methods:**

School-based screening was carried out at primary and secondary schools in the Netherlands with the Strengths and Difficulties Questionnaire (SDQ) self report and teacher report. Additionally, internalizing and psychotic symptoms were assessed with the depressive, somatic and anxiety symptoms scales of the Social and Health Assessment (SAHA) and items derived from the Kiddie-Schedule for Affective Disorders and Schizophrenia (K-SADS). Of 361 Moroccan-Dutch youths (ages 9 to 16 years) with complete screening data, 152 children were diagnostically assessed for psychiatric disorders using the K-SADS. The ability to screen for any psychiatric disorder, and specific externalizing or internalizing disorders was estimated for the SDQ, as well as for the SAHA and K-SADS scales.

**Results:**

Twenty cases with a psychiatric disorder were identified (13.2 %), thirteen of which with externalizing (8.6 %) and seven with internalizing (4.6 %) diagnoses. The SDQ predicted psychiatric disorders in Moroccan-Dutch youth with a good degree of accuracy, especially when the self report and teacher report were combined (AUC = 0.86, 95 % CI = 0.77-0.94). The SAHA scales improved identification of internalizing disorders. Psychotic experiences significantly predicted psychiatric disorders, but did not have additional discriminatory power as compared to screening instruments measuring non-psychotic psychiatric symptoms.

**Conclusions:**

School-based screening for psychiatric disorders is effective in Moroccan-Dutch youth. We suggest routine screening with the SDQ self report and teacher report at schools, supplemented by the SAHA measuring internalizing symptoms, and offering accessible non-stigmatizing interventions at school to children scoring high on screening questionnaires. Further research should estimate (subgroup-specific) norms and optimal cut-offs points in larger groups for use in school-based screening methods.

## Background

While ethnic diversity is increasing in many Western countries, access to youth mental health care is generally lower among ethnic minority youth compared to majority youth [1, 2]. It is unlikely that these lower treatment rates are explained by a lower prevalence of psychiatric disorders, as mental health problems are equally or more prevalent in minority youth as compared to majority youth [3, 4]. Therefore, detection of psychiatric disorders in ethnic minority communities is particularly important to offer timely interventions.

Schools may play an important role in the early detection of psychiatric disorders outside the mental health care system. If school-based screening for psychiatric disorders is effective among ethnic minority youth in Western societies, it might provide a pathway to care for ethnic minority youth and an opportunity to bridge the treatment gap observed in this group.

Since most screening instruments have been developed for Western populations and cross-cultural biases are likely to influence psychometric properties [5], it is not known how these questionnaires can be used in ethnic minority youth. It has been found that construct validity, that is the degree to which the instrument captures the construct to be measured, and factor structure of screening instruments differed between ethnic groups [6, 7]. Using specific questionnaires or underlying factor structures for each subgroup in society is practically unfeasible and undesirable. Instead, subgroup-specific norms may be required [8]. This applies to self-report questionnaires because minority children may interpret questions differently or have different thresholds for reporting psychiatric symptoms, due to language or cultural differences. It also applies to teacher-report questionnaires, as ethnic biases of teachers may influence their ratings, in particular of children from groups with a low social status [9, 10]. As a result of potential cross-cultural biases in construct validity and norms, it is preferable to study the performance of screening instruments for each ethnic group separately.

The Strengths and Difficulties Questionnaire (SDQ) [11] is a questionnaire that is frequently used to screen for psychiatric disorders in children. The ability of the SDQ to detect psychiatric disorders has been shown in community and clinical samples of youth in multiple countries [e.g. 12–14], providing evidence for the applicability of the SDQ in different cultures. Less is known about the test characteristics of the SDQ in ethnic minority youth. In a systematic review on measurement properties of instruments measuring externalizing problems in ethnic minority youth, good internal consistency, content, structural and concurrent validity were found for the SDQ self-report version. For the SDQ teacher report the factor structure was similar in majority and minority groups, whereas norms were likely to be different across ethnic groups [15, 16]. A scoring rule based on the teacher-reported SDQ predicted externalizing disorders equally well in ethnic minority and majority youth in the Netherlands [17]. However, the ability of the SDQ to detect internalizing psychiatric disorders in ethnic minority youth has not been investigated.

Studies report cultural variations in the presentation or symptom expression of internalizing disorders [18]. Therefore, including a wide variety of items on internalizing symptoms may enhance identification of these problems in ethnic minorities. In addition, it has become clear that psychotic experiences in adolescence are important risk markers for severe psychopathology, whether psychotic or non-psychotic [19]. Ethnic minorities have an increased risk for psychotic experiences in childhood [20, 21] and psychotic disorders in adulthood [22], suggesting that psychotic symptoms might be even more important signals of psychopathology in ethnic minority youth. The SDQ includes only five items on internalizing symptoms and no items on psychotic experiences. Therefore, it is clinically relevant to investigate the added value of other screening questionnaires, assessing internalizing and psychotic symptoms, when screening for psychiatric disorders in ethnic minority youth.

The present study was carried out among Moroccan-Dutch youth. In the Netherlands, Moroccan-Dutch youth is the largest ethnic minority population in its age group, has an increased risk to develop childhood psychiatric problems [9] or psychotic disorders in (young) adulthood [23], and is underrepresented in youth mental health care [1]. In addition, Moroccan-Dutch often have a low social status and a relatively wide cultural gap to the majority group, which may increase ethnic and cultural bias in self reports and teacher reports of psychiatric problems [9].

The aim of this study was to examine if school-based screening for psychiatric disorders, using children and teachers as informants, is effective in Moroccan-Dutch youth. We examined the ability of the SDQ to predict any psychiatric disorder, and specific externalizing or internalizing disorders among Moroccan-Dutch youth. Since internalizing disorders are best detected by self-report measures [24] and externalizing disorders by teacher reports [25], both the self-report and teacher-report versions were used. To examine the added value of assessing internalizing and psychotic symptoms, we administered selected scales of the Social and Health Assessment (SAHA) [26] and items adapted for use in a self-report setting from the Kiddie Schedule for Affective Disorders and Schizophrenia (K-SADS) [27]. For each screening instrument we examined the predictive value by comparing the scores on existing (sub)scales to diagnoses of psychiatric disorders.

## Methods

The study had two phases, a screening part and a diagnostic part.

### Participants

#### Screening sample

In the first phase of the study, schools with various educational levels in districts with small and large Moroccan-Dutch populations (range 1.9-9.2 %) were approached, in order to obtain a large sample of Moroccan-Dutch youth with various socio-economic backgrounds. Eight primary schools and ten secondary schools (78.2 %) participated. Children in years six to eight of primary schools (9–12 year olds) and years one to three of secondary schools (12–15 year olds) were included. The overall participation rate was 85.7 %. The total sample consisted of 1563 participants.

According to the ethnic classification of Statistics Netherlands, children were categorized as Moroccan-Dutch when they and one or both parents (first-generation migrants) or when one or both parents (second-generation migrants) were born in Morocco (n = 407).^1^ In case of parents with two different foreign countries of birth, the mother’s country of birth was used to define the child’s ethnic group.

Teachers filled out a questionnaire on 88.7 % of the Moroccan-Dutch children and adolescents (n = 361, see section Measurements).

#### Diagnostic sample

**Fig. 1:**
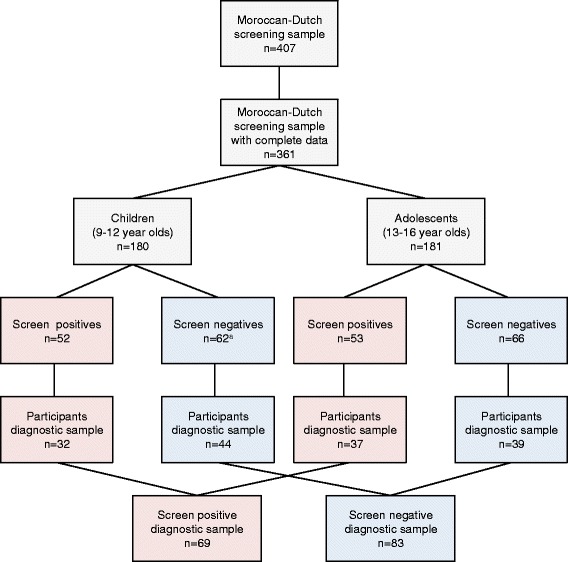
Flow chart of sampling procedure and response. ^a.^ Random selection of 79 screen negative Moroccan-Dutch children

In the second phase of the study, a high-risk and a low-risk subgroup of the total Moroccan-Dutch screening sample were selected for in-depth psychiatric diagnostic assessment. Only youth with complete data (self report and teacher report) were eligible (n = 361). Cut-offs were calculated for children (9–12 year olds, n = 180) and adolescents (13–16 year olds, n = 181) separately. Cut-offs for high-risk and low-risk subgroup selection, were based on scores on nine (sub)scales measuring psychiatric problems: subscales emotional symptoms, conduct problems and hyperactivity of the SDQ self report, subscales conduct problems and hyperactivity of the SDQ teacher report [11], subscales of depressive, somatic and anxiety symptoms scales of the Social and Health Assessment (SAHA) [26] and eight items derived from the Kiddie-Schedule for Affective Disorders and Schizophrenia (K-SADS) [27] assessing psychotic experiences. Moroccan-Dutch youth scoring two standard deviations above the mean of their age category on at least one of the selected (sub)scales (screen positives, n = 105), and Moroccan-Dutch youth scoring below one standard deviation above the mean on all selected (sub)scales (screen negatives, n = 128) were selected for diagnostic evaluation. Of the 233 eligible Moroccan-Dutch youths, 65.2 % (n = 152) participated, 69 were screen positives and 83 were screen negatives. There were no significant differences in response rate between screen positive and screen negative groups (X^2^ = 0.019; df = 1; p = 0.89) or age groups (X^2^ = 0.201; df = 1; p = 0.65). The sampling procedure and response are presented in Fig. 1.

### Procedure

The ethics committee of the VU Medical Centre approved the study. In the screening phase (2009–2010), a letter of introduction and a description of the study were sent to children and parents at their home address in separate envelopes. Parents or primary caregivers additionally received a passive informed consent form, which they could sign and return when they did not want their child to participate. Children had the option to decline at the time the survey was administered. They completed the web-based survey during a regular school day. A trained research assistant introduced the study and at least two research assistants were available in the classroom to answer the children’s questions during administration. Teachers were not involved in the actual administration. For every child, the teacher filled out a paper version of the SDQ teacher report (see section Measurements). All instruments were administered in Dutch.

In the diagnostic phase (2010–2011), the selected Moroccan-Dutch youths and their parents received a letter in Dutch and Moroccan Arabic introducing the study. Parents or primary caregivers were asked to inform the researchers if they refused participation. The remaining families were visited at home in a face-to-face approach. We worked exclusively with female interviewers, because for some Moroccan-Dutch families it is more accepted to welcome unknown females than males into their homes. Additionally, each team consisted of at least one Moroccan-Dutch research assistant to have the option to inform families about the study in Dutch, Moroccan Arabic or a Berber (Tamazight) language. Written informed consent was obtained from all parents and children. Children were interviewed on psychiatric symptoms and impairment by medical doctors trained in transcultural psychiatry using the K-SADS (see section Measurements) during a separate appointment at school or at home. The average time between the screening and diagnostic phase was 13 months.

### Measurements

#### Demographic information

Children filled out questions on demographic characteristics, such as their gender, age, and child’s and parents’ country of birth. A measure of the socioeconomic status of the neighbourhood was obtained from the Netherlands Institute for Social Research [28]. Reading skills were assessed in the diagnostic phase using the One-Minute Reading Task [29].

#### Screening instrument: Strengths and Difficulties Questionnaire (SDQ)

Children and teachers completed the self-report and teacher-report versions of the SDQ [11], consisting of five subscales: emotional symptoms, conduct problems, hyperactivity, peer problems and pro-social behaviour. Each subscale consists of five items on a three-point scale ranging from 0 (*not true*) to 2 (*certainly true*). A *total difficulties score* is generated by summing the scores on four subscales: emotional symptoms, conduct problems, hyperactivity and peer problems (range 0–40). In order to create an aggregated measure of the self report and teacher report, we added the self-report and teacher-report total difficulties scores. Cronbach’s alphas in the screening sample (n = 361) were good for the self report (α = 0.76), the teacher report (α = 0.87), as well as for the aggregated self report and teacher report (α = 0.87) total difficulties scores.

Comparisons of SDQ scores between Moroccan-Dutch youth and other ethnic groups are reported elsewhere [30].

#### Additional screening instruments

##### Social and Health Assessment (SAHA)

Children additionally completed selected scales of the SAHA [26]. The SAHA has been used in ethnically diverse samples in multiple countries [e.g. 31–33]. The *depressive symptoms* scale consists of 15 items, with 11 negative (e.g. ‘I did not feel like eating’, and ‘I felt really down’) and four positive (e.g. ‘I enjoyed doing things’) statements. The *somatic symptoms* scale consists of 12 items representing somatic symptoms commonly reported by children and adolescents (e.g. ‘I felt my health should be better’, ‘I had aches or pains’, and ‘I often woke up early’). Children reported on the presence of depressive symptoms and somatic symptoms during the past month on a three-point scale (0 - not true, 1 - somewhat true, 2 - certainly true). The *anxiety symptoms* scale consists of 13 items (e.g. ‘I worry about what others think about me’, ‘I worry about what is going to happen in the future’, and ‘I stay away from things that make me nervous’). Children reported on the presence of anxiety symptoms on a three-point scale (0 - not true, 1 - sometimes true, 2 - certainly true). By summing the scores of the depressive, somatic and anxiety scales a *total internalizing symptoms* scale was computed. The Cronbach’s alpha of the total internalizing symptoms score in the screening sample (n = 361) was excellent (α = 0.90).

##### Kiddie-Schedule for Affective Disorders and Schizophrenia (K-SADS)

Eight items from the K-SADS [27], adapted for use in a self-report setting, were used to assess the presence of *psychotic experiences*. These items had high resemblance to items that were predictive of adult schizophreniform disorder [34]. Six items assessed delusional experiences (e.g. ‘Have you ever believed that you were being sent special messages through television or radio?’, ‘Have you ever thought you were being followed or spied on?’, and ‘Have you ever believed that you are an important person or have special gifts other people do not have?’). Two items assessed hallucinatory experiences (‘Have you ever heard voices that other people could not hear?’, and ‘Have you ever seen things that other people could not see?’). Responses were made on a three-point scale: 0 – not true, 1 – yes, likely and 2 – yes, definitely and summed into a *psychotic experiences* score. The Cronbach’s alpha of the psychotic experiences score in the screening sample (n = 361) was good (α = 0.76).

Details on the prevalence of psychotic experiences among the Moroccan-Dutch screening sample compared to other ethnic groups have been reported elsewhere [20].

#### Outcome: DSM-IV diagnosis

Children were interviewed using the K-SADS [27], a semi-structured diagnostic interview to assess DSM-IV diagnoses. The interviewers were blind to screening status. For all 41 children and adolescents with decreased functioning in the past year, as operationalized by a score of 7 or lower on the Children’s Global Assessment Scale (C-GAS; included in the K-SADS) [35], summaries of the diagnostic interviews were discussed in consensus meetings of a child psychiatrist (among who TD), a psychiatrist (among who WV), one of the medical doctors who performed the interviews (MA) and a medical student who summarized the audiotapes. The committee, experienced in cross-cultural psychiatry, discussed to formulate one consensus diagnosis per child, based on the scores on the K-SADS, the summary of the audiotape and the clinical evaluation of the medical doctor who conducted the interview. The committee was blind to screening and diagnostic status. An acceptable level of agreement was achieved between the diagnoses made by the interviewers and the committee: the kappa coefficient was 0.76 [36]. The outcome used for analyses was the presence of any, externalizing or internalizing psychiatric disorder according to the DSM-IV criteria, as diagnosed by the interviewers.

### Statistical analysis

Analyses were performed using the Statistical Package for Social Sciences (SPSS), version 20.0. First, demographic characteristics, SDQ, SAHA and K-SADS psychotic experiences scores and numbers of psychiatric disorders in the screening and diagnostic sample were described. There were no significant differences in SDQ, SAHA and K-SADS psychotic experiences scores for migrant status or reading skills. Second, logistic regression analyses were performed to assess the ability of (sub)scales of the used screening instruments to predict a diagnosis of any, externalizing or internalizing psychiatric disorder. Third, Receiver-Operating Characteristic (ROC) analyses were run for the SDQ total difficulties (self report, teacher report and aggregated self report and teacher report), SAHA total internalizing symptoms and K-SADS psychotic experiences scores assessing the diagnostic performance to predict any psychiatric disorder, an externalizing disorder and an internalizing disorder. For all ROC curves the Area Under the Curve (AUC), the optimal cut-off point, based on the cut-off point extending the highest towards the upper left corner, sensitivity and specificity were determined. Finally, the prediction of the optimal cut-off point of the screening instrument with the highest AUC for any psychiatric disorder was shown in a cross-tabulation, with a range of test values. The added value of the SAHA and K-SADS psychotic experiences scales were calculated as well.

## Results

Demographic characteristics and SDQ, SAHA and K-SADS psychotic experiences scores of the Moroccan-Dutch screening and diagnostic sample are presented in Table 1. Boys and girls were represented equally. Most Moroccan-Dutch youths were second-generation migrants and more than half lived in neighbourhoods with a low socioeconomic status in both the screening and the diagnostic sample. A quarter of the children in the diagnostic phase was more than a year behind considering reading skills. The mean age of the screening sample was 12.5 years (SD ± 1.9). During the diagnostic phase of the study, participants were on average one year older (13.6 ± 1.9). SDQ, SAHA and K-SADS psychotic experiences scores and standard deviations were similar in the screening and diagnostic samples.

**Table 1:** Demographic characteristics, SDQ, SAHA and K-SADS psychotic experiences scores and prevalence of psychiatric disorders in the Moroccan-Dutch screening and diagnostic sample

	Screening sample	Diagnostic sample
	(n = 361)	(n = 152)
	% (n)	% (n)
Gender		
Boys	49.6 (179)	49.3 (75)
Girls	50.4 (182)	50 .7 (77)
Migrant status		
First generation	8.0 (29)	6.6 (10)
Second generation	92.0 (332)	93.4 (142)
Neighbourhood socioeconomic status		
Low	52.9 (191)	59.2 (90)
Medium/High	47.1 (170)	40.8 (62)
Reading skills children		
More than one year behind	-	25.7 (39)
Less than one year behind	-	74.3 (113)
Any DSM-IV diagnosis (K-SADS)	-	13.2 (20)
	mean ± SD	mean ± SD
Age	12.5 ± 1.9	13.6 ± 1.9
SDQ self report		
Emotional problems	2.0 ± 2.1	2.2 ± 2.3
Conduct problems	2.3 ± 1.8	2.5 ± 1.9
Hyperactivity	2.7 ± 2.2	2.7 ± 2.3
Peer problems	2.5 ± 1.8	2.6 ± 1.8
Pro-social behaviour	7.9 ± 1.8	7.9 ± 1.8
Total difficulties	9.6 ± 5.5	10.0 ± 6.0
SDQ teacher report		
Emotional problems	1.6 ± 1.8	1.7 ± 1.9
Conduct problems	2.2 ± 2.5	2.4 ± 3.0
Hyperactivity	3.9 ± 3.1	3.9 ± 3.3
Peer problems	1.8 ± 1.7	1.8 ± 1.7
Pro-social behaviour	6.3 ± 2.7	6.3 ± 2.7
Total difficulties	9.5 ± 6.8	9.7 ± 8.0
SDQ self report and teacher report		
Total difficulties	19.0 ± 10.0	19.8 ± 11.9
SAHA		
Depressive symptoms	7.1 ± 4.2	7.4 ± 4.5
Somatic symptoms	4.2 ± 4.6	4.8 ± 5.2
Anxiety symptoms	6.4 ± 5.7	7.1 ± 6.5
Total internalizing symptoms	17.8 ± 11.8	19.3 ± 13.7
K-SADS		
Psychotic experiences	3.1 ± 3.2	3.4 ± 3.4

The prevalence of psychiatric disorders in the Moroccan-Dutch diagnostic sample was 13.2 % (Table 1). Twenty children (28 % of screen positives and 1 % of screen negatives) met the DSM-IV criteria for any psychiatric disorder. Attention-deficit hyperactivity disorder (n = 3, 2.0 %), oppositional defiant disorder (n = 5, 3.3 %) and conduct disorder (n = 5, 3.3 %) were categorized as externalizing disorders (n = 13, 8.6 %). Major depressive disorder (n = 6, 3.9 %) and generalized anxiety disorder (n = 1, 0.7 %) were categorized as internalizing disorders (n = 7, 4.6 %). There was no comorbidity: none of the participants met the diagnostic criteria for more than one DSM-IV diagnosis.

In Table 2, odds ratios for a DSM-IV diagnosis are displayed for each screening instrument. All SDQ, SAHA and K-SADS psychotic experiences (sub)scales significantly predicted a diagnosis of any psychiatric disorder. Further, all SDQ (sub)scales, except for the self-reported emotional symptoms, as well as K-SADS psychotic experiences scale, significantly predicted the diagnosis of an externalizing disorder. The emotional symptoms, hyperactivity and total difficulties scales of the SDQ self report, the emotional symptoms scale of the SDQ teacher report, all SAHA scales and K-SADS psychotic experiences scale all significantly predicted internalizing disorders. No interaction effects for age and reading skills were found for all regression analyses in Table 2.

**Table 2:** SDQ, SAHA and K-SADS psychotic experiences scores as predictors for any, externalizing or internalizing psychiatric disorder (n = 152)

Scale	Any disorder		Externalizing disorder		Internalizing disorder	
	OR	95 % CI	OR	95 % CI	OR	95 % CI
SDQ self report						
Emotional symptoms	**1.37**	**(1.13 – 1.65)**	1.15	(0.93 – 1.44)	**1.59**	**(1.21 – 2.09)**
Conduct problems	**1.29**	**(1.04 – 1.61)**	**1.43**	**(1.11 – 1.84)**	0.98	(0.65 – 1.47)
Hyperactivity	**1.41**	**(1.16 – 1.72)**	**1.37**	**(1.09 – 1.72)**	**1.33**	**(1.00 – 1.78)**
Peer problems	**1.38**	**(1.06 – 1.80)**	**1.56**	**(1.13 – 2.16)**	1.04	(0.68 – 1.59)
Pro-social behaviour	**0.70**	**(0.54 –0.89)**	**0.64**	**(0.48 –0.86)**	0.90	(0.61 – 1.33)
Total difficulties	**1.16**	**(1.08 – 1.25)**	**1.15**	**(1.05 – 1.25)**	**1.13**	**(1.01 – 1.26)**
SDQ teacher report						
Emotional symptoms	**1.55**	**(1.24 – 1.93)**	**1.51**	**(1.18 – 1.92)**	**1.36**	**(1.00 – 1.84)**
Conduct problems	**1.42**	**(1.22 – 1.66)**	**1.67**	**(1.33 – 2.08)**	1.04	(0.82 – 1.33)
Hyperactivity	**1.32**	**(1.13 – 1.53)**	**1.63**	**(1.28 – 2.07)**	0.95	(0.74 – 1.21)
Peer problems	**1.66**	**(1.27 – 2.18)**	**1.88**	**(1.35 – 2.64)**	1.18	(0.78 – 1.77)
Pro-social behaviour	**0.73**	**(0.60 – 0.88)**	**0.69**	**(0.54 – 0.87)**	0.85	(0.64 – 1.13)
Total difficulties	**1.16**	**(1.09 – 1.23)**	**1.22**	**(1.12 – 1.33)**	1.03	(0.94 – 1.12)
SDQ self/teacher report						
Total difficulties	**1.12**	**1.07 – 1.18**	**1.15**	**1.08 – 1.23**	1.05	0.99 – 1.11
SAHA						
Depressive symptoms	**1.15**	**1.05 – 1.27**	1.11	0.99 – 1.24	**1.18**	**1.03 – 1.36**
Somatic symptoms	**1.11**	**1.02 – 1.20**	1.02	0.92 – 1.13	**1.21**	**1.07 – 1.35**
Anxiety symptoms	**1.08**	**1.01 – 1.15**	0.98	0.89 – 1.07	**1.23**	**1.09 – 1.39**
Total internalizing symptoms	**1.05**	**1.02 – 1.08**	1.01	0.97 – 1.05	**1.10**	**1.04 – 1.16**
K-SADS						
Psychotic experiences	**1.25**	**1.10 – 1.43**	**1.22**	**1.06 – 1.41**	**1.22**	**1.01 – 1.46**

Significant differences in bold

**Fig. 2:**
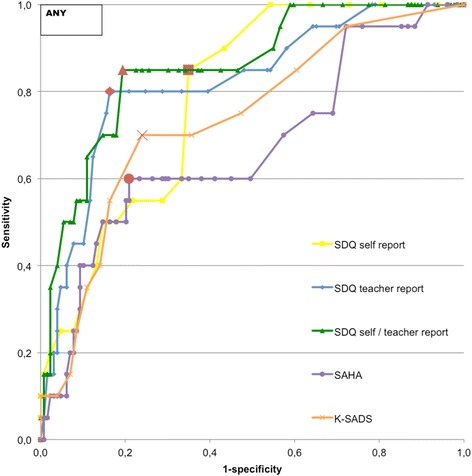
ROC curve predicting psychiatric disorders. Note: Red dots are considered optimal cut-off points. ANY = Any disorder. SDQ self report = SDQ self report; total difficulties, SDQ teacher report = SDQ teacher report; total difficulties, SDQ self/teacher report = SDQ self/teacher report; total difficulties, SAHA = SAHA; total internalizing symptoms, K-SADS = K-SADS; psychotic experiences

**Fig. 3:**
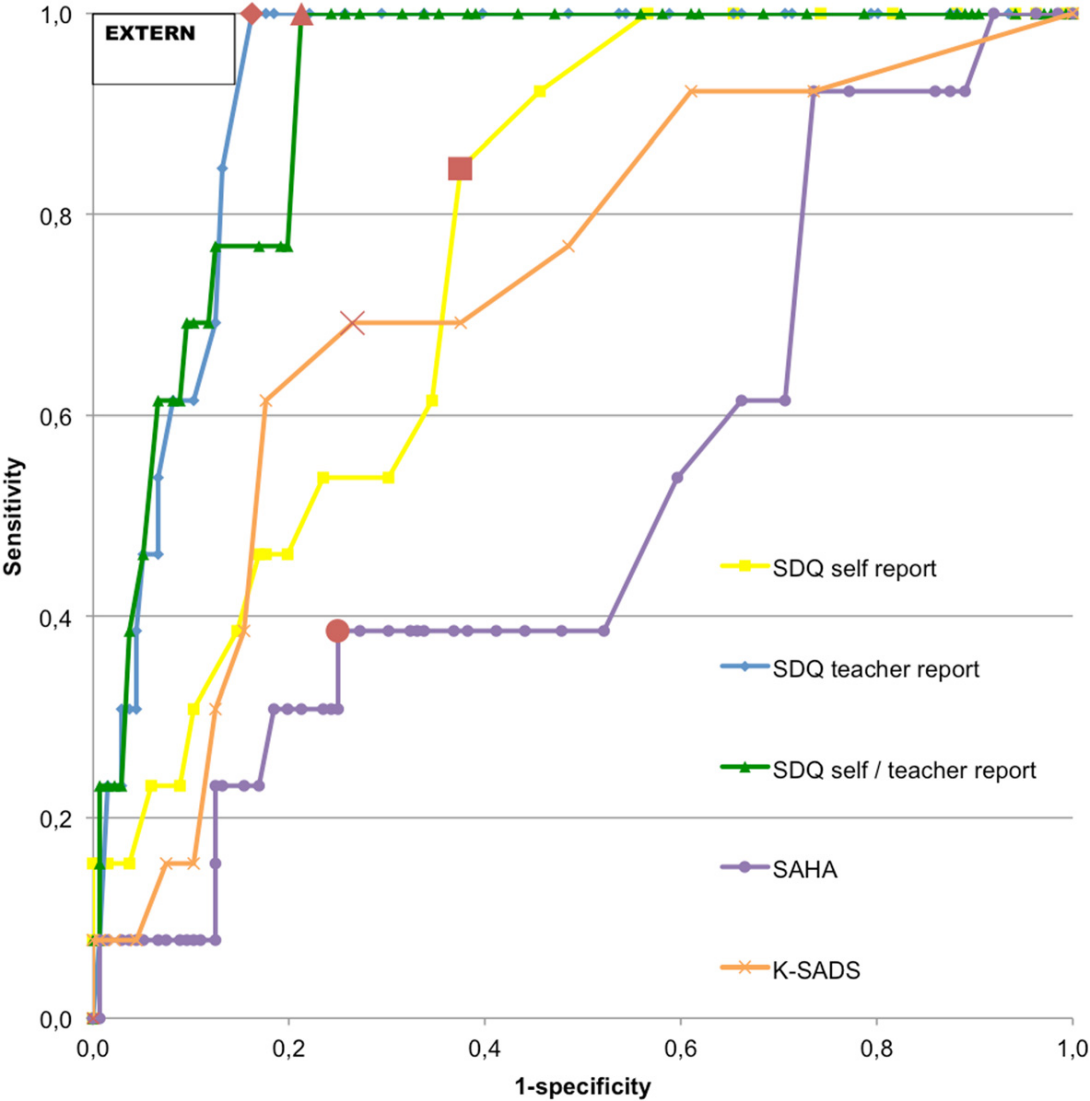
ROC curve predicting externalizing disorders. Note: Red dots are considered optimal cut-off points. EXTERN = Externalizing disorder. SDQ self report = SDQ self report; total difficulties, SDQ teacher report = SDQ teacher report; total difficulties, SDQ self/teacher report = SDQ self/teacher report; total difficulties, SAHA = SAHA; total internalizing symptoms, K-SADS = K-SADS; psychotic experiences

**Fig. 4:**
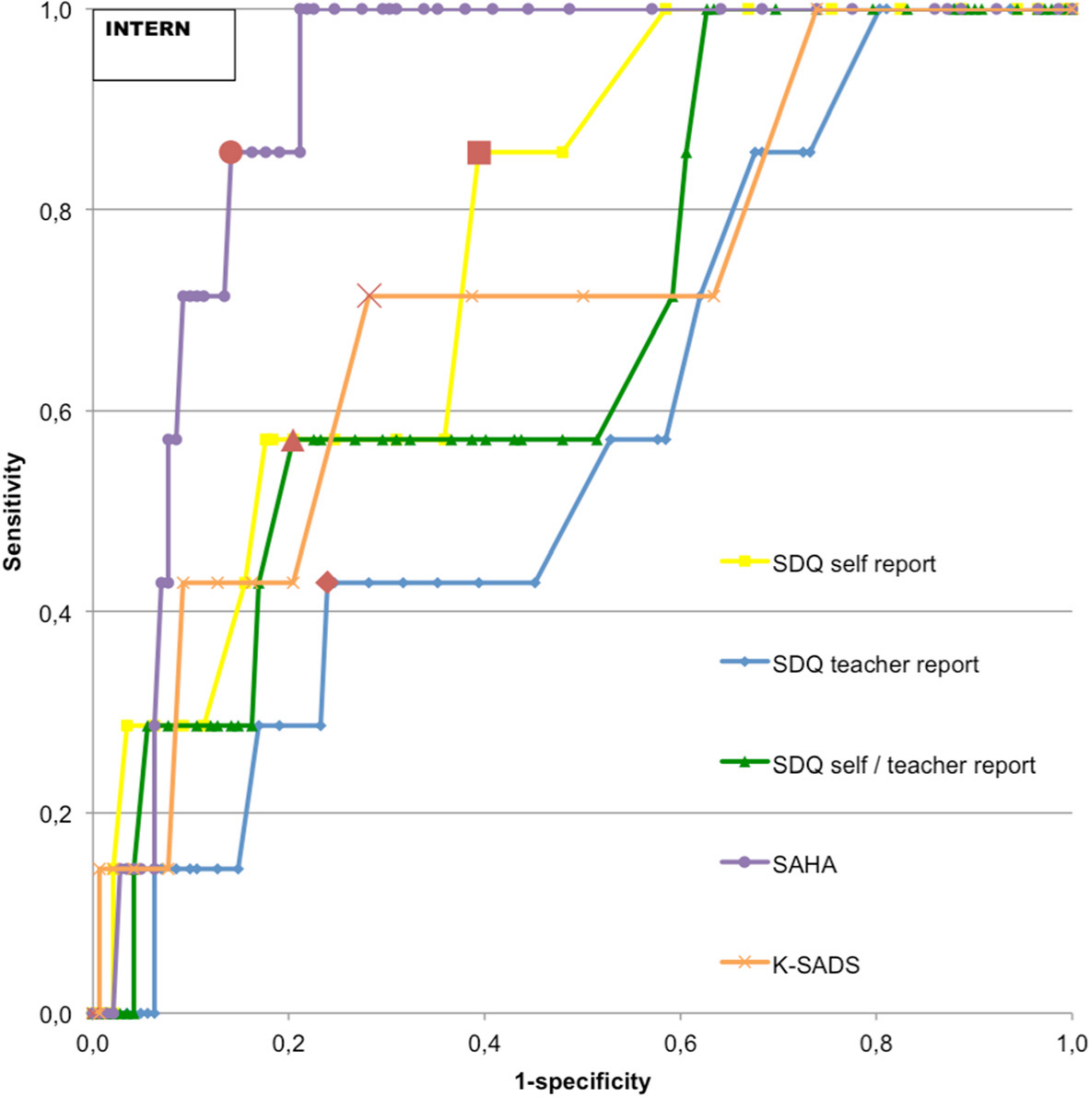
ROC curve predicting internalizing disorders. Note: Red dots are considered optimal cut-off points. INTERN = Internalizing disorder. SDQ self report = SDQ self report; total difficulties, SDQ teacher report = SDQ teacher report; total difficulties, SDQ self/teacher report = SDQ self/teacher report; total difficulties, SAHA = SAHA; total internalizing symptoms, K-SADS = K-SADS; psychotic experiences

ROC curves for all screening instruments predicting the diagnosis of any, an externalizing or internalizing psychiatric disorder are shown in Figs. 2, 3, 4. Table 3 presents all AUC’s, the optimal cut-off points, based on the cut-off point extending the highest towards the upper left corner of the ROC curve (indicated as red dots in Figs. 2, 3, 4), and their corresponding abnormal ranges, sensitivities and specificities.

**Table 3:** Test characteristics of SDQ, SAHA and K-SADS psychotic experiences scores predicting any, externalizing or internalizing psychiatric disorder (n = 152)

	Diagnostic performance	Optimal cut-off^1^	Sensitivity	Specificity
	AUC (95 % CI)	Score (abnormal range)	%	%
Any disorder				
SDQ self report; total difficulties	0.79 (0.70 – 0.88)	10 (10–40)^2^	85	65
SDQ teacher report; total difficulties	0.82 (0.72 – 0.92)	15 (15 – 40)^3^	80	84
SDQ self/teacher report; total difficulties	0.86 (0.77 – 0.94)	26 (26 – 80)	85	80
SAHA; total internalizing symptoms	0.67 (0.53 – 0.81)	25 (25 – 40)	60	79
K-SADS; psychotic experiences	0.74 (0.62 – 0.86)	5 (5 – 16)	70	76
Externalizing disorder				
SDQ self report; total difficulties	0.77 (0.67 – 0.87)	10 (10 – 40)	85	63
SDQ teacher report; total difficulties	0.93 (0.88 – 0.97)	16 (16 – 40)	100	84
SDQ self/teacher report; total difficulties	0.92 (0.87 – 0.97)	26 (26 – 80)	100	79
SAHA; total internalizing symptoms	0.52 (0.36 – 0.69)	25 (25 – 40)	39	75
K-SADS; psychotic experiences	0.73 (0.59 – 0.87)	5 (5 – 16)	69	74
Internalizing disorder				
SDQ self report; total difficulties	0.77 (0.62 – 0.91)	10 (10 – 40)	86	61
SDQ teacher report; total difficulties	0.58 (0.38 – 0.77)	15 (15 – 40)	43	76
SDQ self/teacher report; total difficulties	0.68 (0.50 – 0.87)	30 (30 – 80)	57	80
SAHA; total internalizing symptoms	0.90 (0.85 – 0.96)	33 (33 – 40)	86	86
K-SADS; psychotic experiences	0.71 (0.51 – 0.91)	5 (5 – 16)	71	72

^1^Optimal cut-off points are based on the cut-off point extending the highest towards the upper left corner of the ROC curve

^2^Recommended abnormal range of self-report total difficulties scale of the SDQ in the UK is 18–40 [35]

^3^Recommended abnormal range of teacher-report total difficulties scale of the SDQ in the UK is 16–40 [35]

For any disorder (Fig. 2), all measures significantly outperformed a random predictor (Table 3). A good discriminatory power was achieved with the SDQ aggregated self-report and teacher-report total difficulties score (AUC = 0.86, 95 % CI = 0.77-0.94) and SDQ teacher-report total difficulties score (AUC = 0.82, 95 % CI = 0.72-0.92). For externalizing disorders (Fig. 3), all measures, except the SAHA total internalizing symptoms scale, predicted significantly better than a random predictor (Table 3). The SDQ teacher-report total difficulties score (AUC = 0.93, 95 % CI = 0.88-0.97) and SDQ aggregated self-report and teacher-report total difficulties score (AUC = 0.92, 95 % CI = 0.87-0.97) had excellent discriminatory power. For internalizing disorders (Fig. 4), an excellent discriminatory power was achieved with the SAHA total internalizing symptoms scale (AUC = 0.90, 95 % CI = 0.85-0.96). The SDQ self-report total difficulties score (AUC = 0.77, 95 % CI = 0.62-0.91) and K-SADS psychotic experiences score (AUC = 0.71, 95 % CI = 0.51-0.91) had reasonable discriminatory power (Table 3).

Table 4a shows the prediction of the optimal cut-off point of the screening instrument with the highest AUC for any psychiatric disorder (SDQ self report and teacher report), with the corresponding test values. Using these instruments, a sensitivity of 85 % and specificity of 80 % was reached, 29 % of the screening sample was screen positive. In Table 4b and c the added value of the SAHA and K-SADS psychotic experiences scales are presented. By including the SAHA total internalizing symptoms, the sensitivity increased from 85 to 95 % and the specificity decreased from 80 to 72 %, with an increase from 29 to 37 % of screen positives. By including the K-SADS psychotic experiences the sensitivity increased slightly from 85 to 90 % and the specificity decreased from 80 to 67 %, with a considerable increase of screen positive children from 29 to 41 %.

**Table 4:** Cross-tabulation of the SDQ self report and teacher report prediction of psychiatric disorders and the added value of the SAHA and K-SADS psychotic experiences scales (n = 152)

Screening instrument(s)	Any DSM-IV diagnosis		
a. SDQ self/teacher, cut-off ≥ 26	Diagnosis	No diagnosis	
Screen positive (n = 44)	17	27	Positive predictive value = 39 %
Screen negative (n = 108)	3	105	Negative predictive value = 97 %
Level of the test = 29 %	Sensitivity = 85 %	Specificity = 80 %	Efficiency = 80 %
b. SDQ self/teacher, cut-off ≥ 26	Diagnosis	No diagnosis	
OR			
SAHA, cut-off ≥ 33			
Screen positive (n = 56)	19	37	Positive predictive value = 34 %
Screen negative (n = 96)	1	95	Negative predictive value = 99 %
Level of the test = 37 %	Sensitivity = 95 %	Specificity = 72 %	Efficiency = 75 %
c. SDQ self/teacher, cut-off ≥ 26	Diagnosis	No diagnosis	
OR			
K-SADS, cut-off ≥ 5			
Screen positive (n = 62)	18	44	Positive predictive value = 29 %
Screen negative (n = 90)	2	88	Negative predictive value = 98 %
Level of the test = 41 %	Sensitivity = 90 %	Specificity = 67 %	Efficiency = 70 %

Level of the test = number of screen positives/total number of cases

Efficiency = true positives + true negatives/total number of cases

## Discussion

### Summary of findings

School-based screening using the SDQ predicted psychiatric disorders in Moroccan-Dutch youth with a good degree of accuracy, especially when the self report and teacher report were combined. The additional assessment of internalizing symptoms with the SAHA improved detection of internalizing disorders in Moroccan-Dutch youth. Psychotic experiences predicted both externalizing and internalizing psychiatric disorders, but did not have additional discriminatory power as compared to screening instruments measuring non-psychotic psychiatric symptoms.

### The ability of the SDQ to screen for psychiatric disorders

The SDQ, a screening instrument developed for Western populations, was also effective in the detection of psychiatric disorders in Moroccan-Dutch youth, especially when using both the self report and teacher report. Psychometric properties of screening instruments for psychiatric disorders have frequently been called into question due to cross-cultural biases [5–7]. By showing the good discriminatory power of the SDQ in an ethnic minority group that is marked by a relatively wide cultural gap to the majority group [9], we have provided evidence for the construct validity of the SDQ in this ethnic group. We tested the predictive value of existing (sub)scales, instead of identifying an underlying factor structure in our data. In this way uniformity in the administration might be reached.

Norms may differ substantially between ethnic groups [8]. We compared our cut-offs to normative SDQ data from the United Kingdom (UK), since no Dutch SDQ norms are published and we assumed the UK as a country comparable to the Netherlands. The abnormal range derived from the ROC curve for predicting psychiatric disorders on the teacher report was comparable to the abnormal range recommended in the UK. Zwirs and colleagues also found that similar norms could be used for ethnic minority and majority youth when they developed a scoring rule based on four items of the SDQ teacher report [17]. On the self report, however, the optimal cut-off in Moroccan-Dutch youth was substantially lower than the UK norm (10 versus 16) [37]. This raises questions about the response style of Moroccan-Dutch youth as compared to English youth. Compared to English youth, Moroccan-Dutch youth seem to have a higher threshold to report psychiatric problems [9]. As the difference between the cut-offs is substantial, subgroup-specific norms for Moroccan-Dutch youth on self-report measures should be developed.

### Use of different informants

Consistent with literature on other ethnic groups, internalizing disorders were best detected by self-report measures [24] and externalizing disorders by teacher reports [25]. The discriminatory power improved by combining the scores of the two informants. Without the need to assess parents as a third informant, a satisfactory predictive value was obtained. This not only shows that school-based screening is possible, but also has broader implications. Non-Western parents are less likely to recognize psychiatric problems in their children [38, 39] and are difficult to involve in diagnostic assessments as well as in treatment [e.g. 40]. Our results suggest that information from parents is not necessary for screening or diagnostic purposes for psychiatric problems in Moroccan-Dutch children.

### The added value of the SAHA and psychotic experiences scales

The additional assessment of the SAHA measuring internalizing symptoms increased the accuracy to detect internalizing disorders among Moroccan-Dutch youth. When screening for internalizing disorders, particularly the specificity improved from 61 to 86 % compared to the SDQ self report only. When screening for psychiatric disorders with the SDQ self report and teacher report the sensitivity increased from 85 to 95 % by adding the SAHA; by diagnostically assessing a slightly larger proportion of the screening sample (8 % extra), additional cases of internalizing disorders were found. The SAHA did not predict externalizing disorders, which is interesting because it confirms that the measurement of internalizing symptoms and externalizing symptoms in the screening phase captured different constructs.

Considering the lower discriminatory powers of the psychotic experiences scale and the absent added value to the SDQ self report and teacher report in detecting psychiatric disorders, the additional assessment of psychotic symptoms is not needed for screening purposes. Interestingly though, psychotic experiences were significantly related to self- and teacher-reported psychiatric problems [20], and externalizing and internalizing disorders among Moroccan-Dutch youth. Since such symptoms are an important risk marker for severe psychopathology in adolescence [19] and predictive of psychotic disorders in (young) adulthood [e.g. 34], it is important to take the presence of such symptoms into account in clinical settings.

### Identification of internalizing disorders

Identification of internalizing psychiatric disorders in general screening and diagnostic procedures in ethnic minorities is hampered by cultural variations in presentation or symptom expression [18]. In our sample, the minority of diagnoses was internalizing (4.6 %, 7 out of 152) and the majority externalizing (8.5 %, 13 out of 152). Compared to Dutch prevalence rates for child psychiatric disorders of 8 to 14 %, inter alia constituting of 9 % internalizing and 5 % externalizing disorders [41], the distribution in our Moroccan-Dutch sample is different. In spite of our cultural sensitive approach, we might still have missed cases of internalizing disorders. However, psychiatric symptom profiles in Moroccan-Dutch youth measured with various instruments tend to be more externalizing than internalizing [9, 16, 30], thus it could also be a reflection of the actual distribution.

### Access to care

School-based screening is a practical way to gather multi-informant data of large groups of children, especially when using a web-based survey. A next step is to investigate how to connect children from school-based screening to mental health care. An option is to offer accessible, non-stigmatizing interventions at school to children scoring high on screening questionnaires. If psychiatric problems turn out to be more severe, children should be referred to specialized mental health care centres for further diagnostic assessment and treatment. Such stepped care methods ensure that children detected by screening methods also receive timely intervention. For ethnic minority youth, school-based screening in combination with stepped care interventions may provide an alternative pathway to care and an opportunity to bridge the treatment gap observed in this group.

### Strengths and limitations

The findings of this study are subject to several limitations. First, although studying the performance of screening instruments in one separate ethnic minority group is preferable to overcome possible cross-cultural biases, it precludes generalization of results to other ethnic groups. Second, only Moroccan-Dutch youth scoring very high (above two standard deviations above the mean) or average/low (beneath one standard deviation above the mean) on selected subscales measuring psychiatric problems were included in the diagnostic phase of the study. These cut-off points were chosen to form a group of Moroccan-Dutch youth exhibiting high numbers of internalizing, externalizing or psychotic psychiatric symptoms and to create a contrasting comparison group of Moroccan-Dutch youth with few symptoms on any group of psychiatric symptoms. Since Moroccan-Dutch youths with medium scores were excluded and in these cases it is usually more difficult to make a diagnosis, results on the performance of the screening instruments might be somewhat inflated. Moreover, we did not use the algorithm, based on scores reported by parents, teachers and children, developed for the SDQ for the selection or prediction methods [42]. This was impossible as we did not include parents in our screening method and we had decided to use additional screening questionnaires besides the SDQ and to use the same method for every scale. Third, there was a relatively long time lag between the screening and diagnostic phase. We administered the screening questionnaires only in the screening phase and not in the diagnostic phase. Therefore, we were not able to study stability on the screening instruments. Since childhood and adolescence are turbulent periods, psychiatric symptoms are likely to vary over time. Results can be biased because (i) part of the screen negatives may have developed a psychiatric disorder (test outcome in screening incorrectly negative) and some of the psychiatric problems of screen positive children may have been remitted (higher rate of false positives). Last, medical doctors and not child psychiatrists conducted the diagnostic interviews. The agreement between a committee consisting of psychiatrists and the medical doctors in assigning diagnoses among youth with decreased functioning, indicated that the medical doctors were able to detect psychiatric disorders accurately. However, it is possible that diagnoses were missed. The number of children with a DSM-IV diagnosis was low compared to the prevalence in national sample of Dutch adolescents in 1997 [41]. Diagnoses may also have been missed because only children and not their teachers or parents were interviewed and even more cultural sensitive diagnostic procedures should be used as was discussed earlier.

Strengths of this study were the use of a web-based survey, a high participation rate and multi-informant data on psychiatric problems in a community sample at school in the screening phase of the study, and in-depth diagnostic assessment with a semi-structured psychiatric interview in a subgroup of Moroccan-Dutch youth in the diagnostic phase of the study.

## Conclusions

School-based screening for psychiatric disorders is effective in Moroccan-Dutch youth. We suggest routine screening with the SDQ self report and teacher report at schools, with additional administration of the SAHA measuring internalizing symptoms to enhance identification of internalizing disorders. To make the connection with actual treatment, we suggest offering accessible non-stigmatizing interventions at school to children scoring high on screening questionnaires and referring severe cases to specialized mental health care centres. Further research should estimate (subgroup-specific) norms and optimal cut-off points in larger groups for use in school-based screening methods. School-based screening in combination with timely interventions may reduce the treatment gap observed in Moroccan-Dutch youth in the Netherlands. Other countries with similar underrepresentation of ethnic minority youth in mental health care could possibly benefit in the same way.

## Abbreviations

AUC: Area under the curve
DSM-IV: Diagnostic and statistical manual of mental disorders, fourth edition
SAHA: Social and Health Assessment
SDQ: Strengths and difficulties questionnaire
K-SADS: Kiddie-schedule for affective disorders and schizophrenia
ROC: Receiver-operating characteristic
UK: United Kingdom

## Footnotes

1 This method will have misclassified 1.7 % of Moroccan-Dutch youth as ethnically Dutch, because not their parents, but their grandparents were born in Morocco (third-generation migrants). The third generation of Moroccan migrants in the Netherlands is small [43]
